# Anthropometric and Body Circumference Determinants for Hand Grip Strength: A Population-Based Mon-Timeline Study

**DOI:** 10.1155/2023/6272743

**Published:** 2023-05-30

**Authors:** Agiimaa Byambaa, Indra Altankhuyag, Otgonbayar Damdinbazar, Tsolmon Jadamba, Oyuntugs Byambasukh

**Affiliations:** ^1^Department of Endocrinology, School of Medicine, Mongolian National University of Medical Sciences, Ulaanbaatar 14210, Mongolia; ^2^Department of Division for Science and Technology, Mongolian National University of Medical Sciences, Ulaanbaatar 14210, Mongolia; ^3^TimeLine Research Center, Ayud Tower, Ulaanbaatar 14240, Mongolia; ^4^Brain and Mind Institute, Mongolian Academy of Sciences, Ulaanbaatar 14200, Mongolia

## Abstract

**Background:**

Hand grip strength (HGS) is a tool for diagnosing sarcopenia. In this study, we examined some anthropometric and body circumference measurements as determinants for HGS.

**Methods:**

This cross-sectional study was conducted with participants (Mongolians, *n* = 1080, aged 18–70, mean age of 41.2 ± 13.9 years, 33.7% of men) from the “Mon-Timeline” cohort study. To measure HGS, a digital grip strength dynamometer was used.

**Results:**

Mean HGS in men was 40.1 ± 10.4 kg and in women was 24.5 ± 5.6 kg. Correlation analysis showed that the strongest correlation with HGS was height (*r* = 0.712, *p* < 0.001). Moreover, HGS was inversely correlated with age (*r* = −0.239, *p* < 0.001) and thigh circumference (*r* = −0.070, *p* < 0.01), while it was positively correlated with body weight (*r* = 0.309, *p* < 0.001), neck circumference (*r* = 0.427, *p* < 0.001), upper arm circumference (*r* = 0.108, *p* < 0.0001), lower arm circumference (*r* = 0.413, *p* < 0.0001), and calf circumference (*r* = 0.117, *p* < 0.0001). In the multivariate linear regression analysis (unstandardized B coefficient, 95% CI), age (−0.159, −0.188; −0.129), sex (−9.262, −10.459; −8.064), height (0.417, 0.357; 0.478), lower arm circumference (1.003, 0.736; 1.270), and calf circumference (−0.162, −0.309; −0.015) were significantly associated with HGS.

**Conclusions:**

When detecting sarcopenia using HGS, it is important to take into account variables such as body height and body circumference.

## 1. Introduction

Muscle deficiency (sarcopenia) is a sign of not only malnutrition but also aging [1]. The overall estimate of sarcopenia prevalence was 10% in 2017, based on a meta-analysis of 58,404 participants aged 60 in 115 studies [2]. As well, sarcopenia and presarcopenia, linked to obesity and malnutrition rather than aging, affect up to 5% of people under the age of 60 [1, 3]. Numerous studies have shown that the risk of premature mortality is higher in those with sarcopenia [4]. As a result, recent review articles have emphasized the importance of the need for screening and early diagnosis of sarcopenia [4, 5]. Xu et al. noted that the burden of sarcopenia on health is independent of the definition of sarcopenia and of any population [4]. The widely used method in clinical practice is BMI and is not able to distinguish body fat and muscle mass [6]. Determining the precise ratio of human body composition using imaging techniques and laboratory methods can be both costly and time-consuming [7].

Numerous studies have shown that measuring the hand grip strength (HGS) can predict muscle function over a short period of time [8–12]. In addition to examining the value of the HGS, reference studies continue to examine the factors or hypotheses that influence it [9–13]. For instance, a study found that HGS was strongly correlated with body height, and they developed reference values for HGS/height ratio [12]. In a UK Biobank study, HGS/height was one of the main biomarker determinants of mortality from chronic diseases [14]. Few studies have been conducted on the correlation between HGS and body circumstances [9, 10, 13]. Because sarcopenia is a skeletal muscle deficiency, we hypothesize that HGS is correlated with limb circumference. In this study, we examined body circumstances, as well as body height and weight, to determine which correlations were important in predicting HGS. It is hoped that further studies will provide important information on the importance of body circumstances in the detection of sarcopenia, as well as in sarcopenia using the value of the HGS.

## 2. Materials and Methods

### 2.1. Data Source

This study was conducted with participants from the “Mon-Timeline” study of the Mongolian National University of Medical Sciences [15]. “Mon-Timeline” is the name of a multidisciplinary, population-based, prospective cohort study conducted in Mongolia to investigate various health problems among Mongolians, especially those associated with oral, psychological, mental, and neurocognitive health problems in 2020. This study established a national database based on the data collected (*n* = 2709, aged between 13 and 70 years) from urban and rural regions in Mongolia. The study design and recruitment processes are described in detail elsewhere [15].

In this study, we included people aged 18 years and over (*n* = 1839) in this study. The exclusion criterion was missing data of HGS and anthropometry measures (*n* = 536). In addition, participants were athletes or with a restriction of movement in the upper extremities such as neuromuscular diseases and injury (*n* = 89). People who answered “poor” to a question to assess their health were excluded from the study because they may have a chronic illness (*n* = 52). We also excluded outlier data of HGS and anthropometry measures (*n* = 82). Finally, a total of 1080 participants were included in the current analyses. From the cohort data, we collected age, sex, education level, and body measurements (described below). Education was categorized as low (from no education to preuniversity education) and high (higher vocational education and university).

### 2.2. Hand Grip Strength Measurement

To measure HGS, a digital grip strength dynamometer (factory standardized and certified) was used that was made in Japan (Takei Hand Grip Dynamometer 5401-C) [16]. The instrument is capable of measuring from 5 to 100 kg and the measurements are recorded with 0.1 kg accuracy. During the measurement, all participants stood up straight on their feet with shoulder width apart, with fully extended elbows, bent hand fingers at 90° and gripped once with each hand. Participants were advised to grip to full strength for at least 3 seconds and while gripping not to move the dynamometer or hold breath. The HGS was measured in both hands of the study participants. We used the dominant HGS based on the domination of the hands that were self-reported or the highest measurement of all hands.

### 2.3. Anthropometry and Circumference Measurements

Participants' body weight (in kg), height (in cm), and body circumferences such as neck, chest, midarms (upper arm circumference), forearm (lower arm circumference), waist, hip, thigh, and calf circumferences were measured by well-trained assistants implementing a standardized protocol. The neck circumference was measured under the laryngeal prominence and from the middle point between the base of the neck and the upper part of the sternum. The chest circumference was measured at the widest point. To measure the circumference of the upper arm circumference, take a point in the center of the line drawn between the prosessus.acromion and procsessus.olecranon and bend the elbow joint 90°. For the circumference of the forearm, the palm was pointed upwards and the largest portion of the forearm was measured. Waist circumference was measured on bare skin at the natural indentation between the 10th rib and the iliac crest. When there was no indentation, we measured it in the middle between the navel and rib cage. Hip circumference was measured from a distance around the largest part of the hip. To measure thigh circumference, the weight of the body was concentrated on the other leg to relax the thigh muscles, measuring 2 cm below the hip joint. The calf circumference was measured at the widest point between the knee and ankle joints, across the m. calf of the tibia [17, 18].

Body mass index (BMI; kg/m^2^) was subsequently calculated. A semiautomatic device was used to measure blood pressure in a half-sitting position.

Blood pressure was measured by well-trained assistants implementing a standardized protocol with an automated device (Pangao, PG-800B69, The Hague, the Netherlands) in a quiet room with room temperature after 10 minutes' rest in the supine position. The size of the cuff was chosen according to the arm circumference.

### 2.4. Statistical Analysis

We conducted normality tests on all continuous variables using both the Shapiro–Wilk test and visual inspection of histograms. Our analysis showed that all variables followed a normal distribution. Descriptive statistics for the general characteristics of the study population were expressed as means with standard deviation (SD), while categorical variables were expressed as numbers with percentages. The differences among groups were compared using Student's *t*-test and Pearson's chi-square test. We used Pearson's correlation to study the association between HGS and anthropometry and circumference measurements. To define predictors for HGS, linear regression analysis was used. After univariate regression analysis, we performed a multivariate linear regression analysis on the variables which had a value of *p* < 0.05.

For all statistical analyses, we used IBM SPSS V.28.0 (IBM, Chicago, IL) and GraphPad Prism V.9.0 (GraphPad Software, La Jolla, CA). A statistical significance level was set at *p* < 0.05 for all tests.

## 3. Results

The mean age was 41.2 ± 13.9 years. 33.7% (*n* = 364) of the study participants were men. There were no significant differences in the mean age and blood pressure according to sex. Moreover, the percentage of the age group and education level was not significantly different for men and women. Anthropometric measurements showed no significant differences in chest, upper arm circumference, calf, or waist circumference (Table 1).

**Figure 1:**
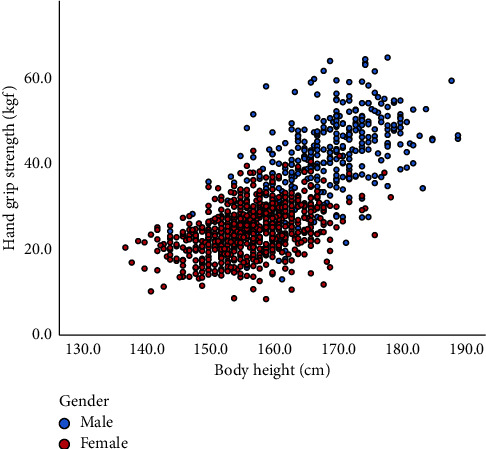
Correlation between body height and dominant hand grip strength.

Mean HGS in men was 40.1 ± 10.4 kg and in women 24.5 ± 5.6 kg (*p* < 0.001). Correlation analysis showed that the strongest correlation with HGS was height (*r* = 0.712, *p* < 0.001, Figure 1 and Table 2).

There was a tendency for HGS to decrease with age (*r* = −0.239, *p* < 0.001). Indicators of relative muscle development, such as the circumference of the forearm and calf, are positively correlated with HGS (Table 2). Furthermore, there was a tendency, with a BMI and thigh and hip circumference increase leading to obesity-associated HGS decreases.

As shown, the variables in Table 3, which had a value of *p* < 0.05 in the univariate linear regression analysis, were tested in a multivariate linear regression analysis. Among them, age, sex, body height, lower arm circumference, and calf circumference were significantly associated with HGS.

Finally, we made an equation for the HGS using determinant variables: Hand grip strength = (−40.953) + age [year] × (−0.154) + body height (cm) *x* 0.407 + lower arm circumference (cm) × 1.005 + sex (men = 0, women = 1) × (−9.526) + calf circumference (cm) × (−0.191); corrected *R*^2^ = 0.68, *p* < 0.0001.

## 4. Discussion

This study examined the predictive determinants of hand grip strength concerning anthropometric and body circumference measurements. In addition to other studies where they found height is the main predictor of HGS, we found body circumferences such as lower arm and calf circumferences are crucial determinants of HGS.

According to our hypothesis, HGS depended on a person's height. In 2019, the Asian Sarcopenia Working Group examined and concluded that when muscle mass is adjusted for BMI, it is better than that adjusted for height in the prediction of comorbidities [19]. Consequently, we also considered BMI to be a more predictive factor than height for HGS, but this was not supported in our study. In accordance with the results of our study, some studies have found that body height is strongly associated with HGS [11, 12, 14]. For instance, a study by Rita S observed that the relationship between HGS and nutritional status is affected by body height, and the HGS is closely related to height [11]. Wendy's study also found HGS was significantly correlated with body height, and they developed a reference value for the index of handgrip, which is HGS adjusted by squared of height, suggesting that the HGS/height ratio could be a significant predictor of disease [12]. The UK Biobank Cohort Study identified the main biomarker determinants of biomarker age, which is related to chronological age. The study found that grip strength/height is one of the ten main predictors of mortality from chronic diseases and age-related hospitalization [14].

Sarcopenia is a skeletal muscle deficiency; thus, our study hypothesis was that the HGS would correlate with all length of the limb circumference. Before the multivariate regression analysis, the univariate regression analysis described the calf and thigh circumferences were inversely related with the HGS, and the arm circumferences were positively correlated with HGS according to our hypothesis. However, the multivariate regression analysis showed that only calf and lower arm circumference were associated with HGS. Numerous previous studies have shown that most of the circumferences of the body such as the neck, the upper and lower circumferences, the circumferences of the thighs and calves are related to the hand grip strength [8, 20–22]. But not many of them were associated with sarcopenia [8, 21]. For instance, in the study of Ishii, the circumference of the calf was statistically significant, but the arm circumferences were not significant in the detection of sarcopenia [8]. However, these indicators have the same statistical significance in terms of HGS. For instance, the correlation coefficients between calf circumference and HGS and lower arm circumference and HGS are the same, 0.35 for men (*p* < 0.0001) and 0.33 and 0.21 for women (*p* < 0.0001), respectively. In this study, as statistical models, HGS and calf circumference are better predictors of the development of sarcopenia, while a model including HGS, and arm circumference was not significant. Therefore, some parameters of the body circumference may be indicators of sarcopenia like HGS, while others may be related to the muscles involved in the force of the handle rather than muscle loss. In addition, studies have shown that a decrease in muscle mass during sarcopenia can be indicated by a decrease in the circumference of the calf [8, 23], and that a decrease in muscle mass can be indicated by subcutaneous fat around the upper arm and the circumference of the upper arm [24].

As mentioned previously, some parameters of the body circumference can be related to the strength of the handle because they are related to the muscles involved in the strength of the handle rather than muscle deficiency. Some studies have also found that arm length and arm width are important predictors in the measurement of HGS [25–27]. Similar to the results of our study, other studies have shown that in addition to the length of the hand, the forearm circumference is an important indicator of the force of the handle [28]. Therefore, it is necessary to pay attention to parameters such as arm circumference or the size of the hand itself, and whether it is important to detect sarcopenia.

The strength of our study is that the various body circumferences are studied in relation to HGS which will provide important information for further studies. This is because it is important to find a simple method, such as measuring HGS, which can be used clinically to detect muscle deficiencies caused by obesity. Because BMI cannot be measured in terms of detecting muscle mass [6, 7], and it is important to screen people with sarcopenic obesity who are more likely to develop CVD than obese patients with sarcopenia [3–5]. In our study, HGS was inversely related to obesity indicators such as BMI and waist circumference and has shown that it is possible to detect muscle deficiency due to obesity by measuring HGS. However, the limitation of our study is that it involves relatively few men and does not separate statistics by sex. However, this followed a method of combining both sexes in the study of Rodríguez-García and did not consider it necessary to analyze the sexes separately [12]. Another limitation of this study is that age stratified analysis was not performed. In the literature, age is the main predictor of HGS that was also observed in our study, and hand grip strength decreased with age [29–31]. This was also noted in our previous study, where it was considered appropriate to have different references to age groups [16]. However, we aimed to explore various body circumferences that are related to HGS through a multivariate regression analysis including age. In addition, regression analysis for each age group was not appropriate to estimate the numerical value of age predictors differently across age groups because we have included relatively young individuals in this study. Finally, as age increases, the predictive value for age can be higher. It is therefore important for future research to explore the numerical value of the difference in predictive value by the age group.

## 5. Conclusions

We found that age, sex, body height, and lower arm circumference are determinant factors for HGS. In particular, few studies have shown that the relationship between arm circumference and HGS is important, and we have found that it is a predictor of HGS. As follows, in the future, it is necessary to study whether the arm circumference remains an important parameter for the detection of sarcopenia using HGS, as well as for the detection of sarcopenia by other methods.

## Figures and Tables

**Table 1:** Characteristics of the study population.

Findings	Men (*n* = 364)	Women (*n* = 716)	*P* value
Age (years)	40.9 ± 14.7	41.4 ± 13.7	0.605
<40 years, % (*n*)	44.7 (163)	43.6 (312)	0.392
>40 years, % (*n*)	55.2 (201)	56.4 (404)	
Education: Low level, % (*n*)	41.2 (150)	46.9 (336)	0.056
Systolic BP (mm Hg)	125.8 ± 16.2	123.8 ± 17.4	0.281
Body weight (kg)	73.8 ± 15.0	66.9 ± 14.1	**<0.0001**
Body height (cm)	167.9 ± 7.9	156.5 ± 6.3	**<0.0001**
Body mass index (kg/m^2^)	26.1 ± 5.0	27.2 ± 5.5	**0.002**
Neck circumference (cm)	37.4 ± 3.7	33.5 ± 2.8	**<0.0001**
Chest circumference (cm)	95.2 ± 10.6	95.9 ± 11.4	0.346
Upper arm circumference (cm)	29.2 ± 3.8	29.0 ± 4.2	0.497
Lower arm circumference (cm)	26.0 ± 2.5	24.2 ± 2.4	**<0.0001**
Thigh circumference (cm)	50.6 ± 6.3	53.9 ± 7.0	**<0.0001**
Calf circumference (cm)	35.4 ± 3.3	35.3 ± 3.9	0.633
Waist circumference (cm)	90.0 ± 14.7	88.2 ± 14.7	0.055
Hip circumference (cm)	97.4 ± 9.1	99.7 ± 10.1	**<0.0001**

*Notes*. BP, blood pressure. Student's *t*-test and Pearson's chi-square test were used. Significant differences are indicated by bolded *p* values (<0.05).

**Table 2:** Correlation between anthropometric measures and dominant hand grip strength.

Variables	*r*	*P* value
Age (years)	−0.239^*∗∗*^	**<0.0001**
Body weight (kg)	0.309^*∗∗*^	**<0.0001**
Body height (cm)	0.712^*∗∗*^	**<0.0001**
Body mass index (kg/m^2^)	−0.055	0.071
Neck circumference (cm)	0.427^*∗∗*^	**<0.0001**
Chest circumference (cm)	0.028	0.366
Upper arm circumference (cm)	0.108^*∗∗*^	**<0.0001**
Lower arm circumference (cm)	0.413^*∗∗*^	**<0.0001**
Thigh circumference (cm)	−0.070^*∗*^	**0.021**
Calf circumference (cm)	0.117^*∗∗*^	**<0.0001**
Waist circumference (cm)	0.038	0.215
Hip circumference (cm)	−0.014	0.647

*Notes*: Pearson correlation. Significant differences are indicated by bolded *p* values (<0.05). Additionally, the level of statistical significance was indicated as ^*∗∗*^*p* < 0.01 and ^*∗*^*p* < 0.05.

**Table 3:** Multiple linear regression of anthropometric determinants for hand grip strength.

Variables	*ß* coefficient	95% CI		*P* value
		Lower limit	Upper limit	
Age (years)	−0.159	−0.188	−0.129	**<0.0001**
Sex (women)	−9.262	−10.459	−8.064	**<0.0001**
Body weight (kg)	−0.032	−0.092	0.029	0.306
Body height (cm)	0.417	0.357	0.478	**<0.0001**
Neck circumference (cm)	0.075	−0.100	0.249	0.400
Upper arm circumference (cm)	0.066	−0.117	0.250	0.477
Lower arm circumference (cm)	1.003	0.736	1.270	**<0.0001**
Calf circumference (cm)	−0.162	−0.309	−0.015	**0.031**
Thigh circumference (cm)	−0.018	−0.097	0.060	0.647

*Notes*: Multiple linear regression. Significant associations are indicated by bolded *p* values (<0.05).

## Data Availability

The data used to support the findings of this study are available from the corresponding author upon request.
